# Current Trends of Artificial Intelligence for Colorectal Cancer Pathology Image Analysis: A Systematic Review

**DOI:** 10.3390/cancers12071884

**Published:** 2020-07-13

**Authors:** Nishant Thakur, Hongjun Yoon, Yosep Chong

**Affiliations:** 1 Department of Hospital Pathology, Yeouido St. Mary’s Hospital, College of Medicine, The Catholic University of Korea, 10, 63-ro, Yeongdeungpo-gu, Seoul 07345, Korea; nishantbiotech2014@gmail.com; 2 AI Lab, Deepnoid, #1305 E&C Venture Dream Tower 2, 55, Digital-ro 33-Gil, Guro-gu, Seoul 06216, Korea; hyoon@deepnoid.com

**Keywords:** artificial intelligence, pathology, colorectal neoplasm, deep learning, systematic review

## Abstract

Colorectal cancer (CRC) is one of the most common cancers requiring early pathologic diagnosis using colonoscopy biopsy samples. Recently, artificial intelligence (AI) has made significant progress and shown promising results in the field of medicine despite several limitations. We performed a systematic review of AI use in CRC pathology image analysis to visualize the state-of-the-art. Studies published between January 2000 and January 2020 were searched in major online databases including MEDLINE (PubMed, Cochrane Library, and EMBASE). Query terms included “colorectal neoplasm,” “histology,” and “artificial intelligence.” Of 9000 identified studies, only 30 studies consisting of 40 models were selected for review. The algorithm features of the models were gland segmentation (*n* = 25, 62%), tumor classification (*n* = 8, 20%), tumor microenvironment characterization (*n* = 4, 10%), and prognosis prediction (*n* = 3, 8%). Only 20 gland segmentation models met the criteria for quantitative analysis, and the model proposed by Ding et al. (2019) performed the best. Studies with other features were in the elementary stage, although most showed impressive results. Overall, the state-of-the-art is promising for CRC pathological analysis. However, datasets in most studies had relatively limited scale and quality for clinical application of this technique. Future studies with larger datasets and high-quality annotations are required for routine practice-level validation.

## 1. Introduction

Colorectal cancer (CRC) is one of the most common cancers and the second leading cause of mortality worldwide. More than one million new CRC cases and 551,269 CRC-related deaths have been reported in 2018 [1]. Although the incidence is still higher in Western countries [2], it is also increasing in developing countries due to rapid adoption of the urban lifestyle. In China and India, the incidence of CRC is projected to double by 2025 as compared to that in 2002 [3]. Early diagnosis is an important step in reducing CRC mortality, and colonoscopy is one of the most common and powerful screening methods [4]. However, the number of colonoscopy examinations is enormously increasing [4], and microscopic examination is becoming more labor-intensive and time-consuming. In addition, the pathological diagnosis of colonoscopy biopsy samples can be easily biased by individual pathologists’ experience and knowledge, leading to inter-observer and intra-observer variations [5]. For these reasons, a gap exists between the pathological standards for the diagnosis of colon lesions in Western countries (often referred to as the Vienna classification) [6] and Eastern countries (also known as the Japanese classification) [7]. Western pathologists diagnose cancer based on the presence of invasion through the muscularis mucosa into the submucosa, while Eastern pathologists do so based on nuclear and architectural changes of the cells [8]. Therefore, there is a need for a more standardized or unified method that can also reduce the confusion among specialists.

Recently, artificial intelligence (AI) has made significant progress and has shown promising results in the medical field, such as in radiologic diagnosis [9], disease diagnosis through gross and microscopic images [10], drug development [11], and precision oncology [12]. Previous evidence showed that a convolutional neural network (CNN)-based model has shown excellent results in tumor detection, classification, gland segmentation, and grading, especially for breast [13], brain [14], lung [15], gastric [16], ovarian, and prostate [17,18] cancers. In addition, there have been various attempts to apply AI in the pathologic image analysis of CRC. A recent study conducted by a Japanese group showed relatively good accuracy of an e-pathologist software program when compared with that of human pathologists in terms of classifying the tumor into four categories: adenoma, carcinoma, no neoplasia, and unclassifiable [19]. More recently, a study conducted by a German group showed that a deep learning-based model could classify the human tumor microenvironment and predict the survival of CRC patients using histologic images [20]. Upon closer observation; however, each study had many limitations such as use of annotated data with limitations, relatively small number of training and validation datasets for generalization, and impaired study design for an appropriate level of evidence. Another technical barrier in the AI development, especially for CRC, is the presence of various artifacts in CRC samples, such as cauterization. Although the development of an AI system in CRC is gaining the attention of scientists, and an increasing number of studies are being published, no appropriate review has been published thus far.

Therefore, we performed a systematic review including qualitative and quantitative analyses of the current application of deep learning in CRC pathology image analysis.

## 2. Results

### 2.1. Eligible Studies and Characteristics

**Figure 1:**
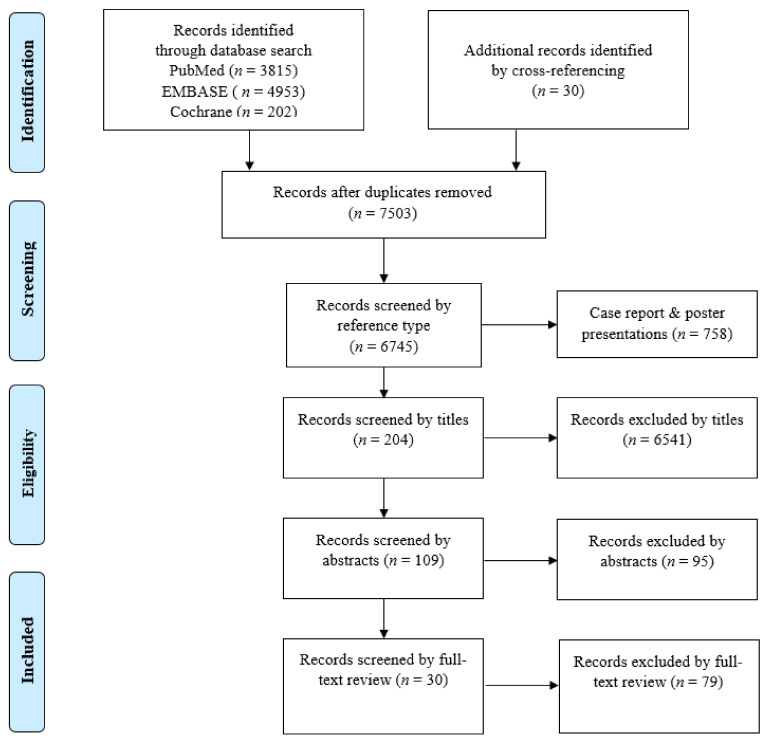
Flow diagram of the study selection process.

A flow diagram of the literature selection process is presented in Figure 1. In total, 9000 records were identified in the initial search (3815 in PubMed, 4953 in EMBASE, 202 in the Cochrane library) and 30 records were identified by cross-referencing. After excluding 1497 duplicates, a total of 7503 records were screened by the type of reference. After 758 records of case reports and poster presentations were excluded, 6745 records were screened by their titles. After 6541 records were excluded, 204 records were screened by their abstracts. After 95 records were excluded, 109 records were screened by full-text review. After excluding 79 records, only 30 records, covering 40 deep learning models, were deemed eligible for an in-depth review and qualitative and quantitative analyses.

### 2.2. Applications of Deep Learning in Colorectal Cancer Pathology Image Analysis

**Figure 2:**
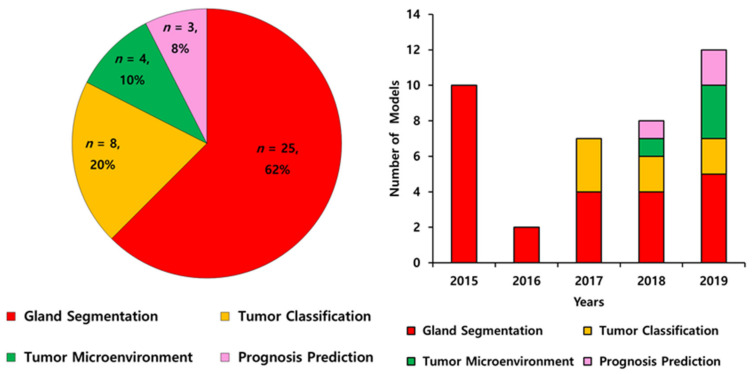
The algorithm features of artificial intelligence in pathological image analysis of colorectal cancers from 2015 to 2019 (**A**) and annual trends (**B**).

The literature according to the deep learning models applied in colorectal cancer are summarized in Table 1 and Table 2. All 40 included models were published in 30 journal articles between 2015 to 2020 and conducted globally (23 models in Europe, four models in the USA, one model in Canada, one model in Russia, and 11 models in Asian countries). The algorithm features of the models were gland segmentation (*n* = 25, 62%), tumor classification (*n* = 8, 20%), tumor microenvironment characterization (*n* = 4, 10%), and prognosis prediction (*n* = 3, 8%) (Figure 2A). The number of published models has increased (Figure 2B).

#### 2.2.1. Gland Segmentation

In the gland segmentation category, 25 models were found, and all the models used 165 images of the Warwick QU dataset from the Gland Segmentation in Colon Histology Images (GlaS) challenge in 2015 (Table 1) [21,22,23,24,25,26,27,28,29,30,31,32,33,34,35,36,37]. The GlaS challenge was a sub-event of the 18th International Conference on Medical Image Computing and Computer-Assisted Intervention (MICCAI) held to solve the problem of gland segmentation [50]. As a result, 110 teams participated in the challenge, and the performance of the top 10 models from the top six teams were available for qualitative analysis. The performance metrics were as follows: F1-score (accuracy of the detection of each gland, ranging from 1 to 100; the higher, the better), object Dice (agreement between two sets of the object, ranging from 1 to 100; the higher, the better), and object Hausdorff (borderline-based analogy between two sets of samples: the closer to 1, the better). Overall, the teams showed F1-scores up to 0.91, object Dice scores up to 0.89, and object Hausdorff scores up to 45.4 in the offsite dataset (Part A) and similar results in the onsite dataset (Part B) (Table 1) [21,22]. All the studies after the GlaS challenge also used the same dataset and showed better performance: F1-scores up to 0.92, object Dice scores up to 0.91, and object Hausdorff scores up to 39.8 for Part A as well as Part B (Table 1) [23,24,25,26,27,28,29,30,31,32,33,34,35,36,37].

**Figure 3:**
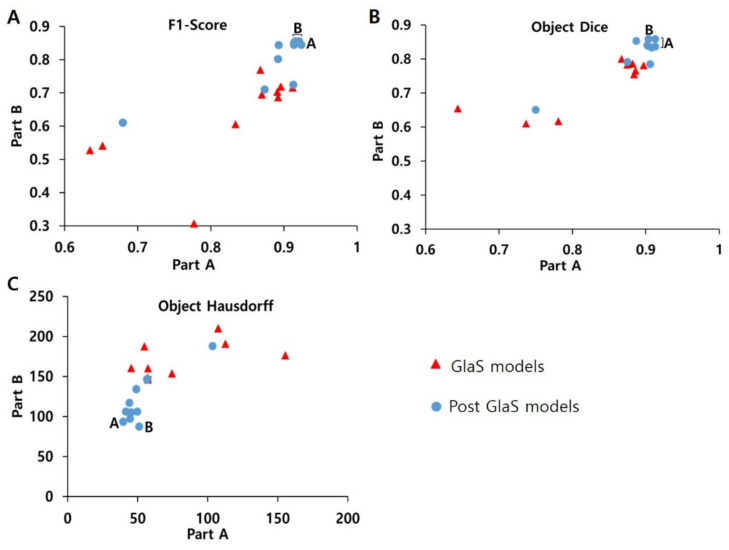
Comparison between (**A**) F1-score, (**B**) object Dice, (**C**) object Hausdorff of GlaS models, and Post-GlaS models. Part A means data used as off-site and Part B data used as on-site and A and B indicate the best model for Parts A and B, respectively. The models by Yan, Yang, and Zhang et al. show the best F1-scores, the models by Yang, Ding, and Graham show the best object Dice, the models by Ding and Manivannan et al. show the best object Hausdorff.

In the quantitative analysis comparing GlaS and post-GlaS studies, post-GlaS studies showed better accuracy in both Parts A and B (Table 3 and Figure 3). According to the rank-sum analysis of the 20 models, the model proposed by Ding et al. in 2019 showed the best general performance of all [33]. For individual parameters, other models proposed by Yan [30], Yang [25], and Zhang [27] et al. showed the best F1-score. The models proposed by Yang [25] and Graham [29] et al. showed the best object Dice, and the models proposed by Manivannan et al. [31] showed the best object Hausdorff (Table 3 and Figure 3).

#### 2.2.2. Tumor Classification

In the tumor classification category, there were eight models, most of which were designed to classify the dataset into two to six specific categories, mostly according to tumorigenic stages such as normal, benign hyperplasia, adenoma (also called intraepithelial neoplasia or precancerous lesion), adenocarcinoma, etc., or histologic subtypes of colonic polyps or adenocarcinomas (Table 2) [38,39,40,41,42,43,44,45]. The size of the datasets varied greatly from 30 to 154,302 patches (27–4036 whole slide images (WSIs)). Most studies did not describe in detail the annotation process such as the number of participating specialists, which represent the quality of the dataset. Considering that at least 2000 datasets per class are generally suggested for the classification models, the dataset size of most studies was insufficient for a high level of evidence. Because each model has an individual focus and datasets with different characteristics, a direct comparison between models was meaningless and impossible. Thus, the absence of a standardized dataset or classification system was a major limitation of all the studies. Although most studies showed good performance with generally high sensitivity (up to 95.1%), specificity (up to 92.7%), area under the curve (AUC, up to 0.99), and accuracy (up to 99.2%), the evidence level of each model seemed to be not high enough because of the aforementioned limitations.

#### 2.2.3. Tumor Microenvironment Analysis

In tumor microenvironment analysis category, four models were included, each of which used a different strategy to identify the CRC microenvironment [20,46,47,48]. Three models focused on the classification of the cell types (into four to nine cell types, such as epithelial, inflammatory, fibroblast, and others or adipose, background, debris, lymphocytes, mucus, smooth muscle, normal mucosa, stroma, and cancer epithelium) [20,46,48], while another model focused on the detection of immune cells (CD3^+^ and CD8^+^ cells) using the immunohistochemistry (IHC) [47]. The results of each study presented varying outcomes using different kinds of performance metrics. Although it is not easy to simplify the performance of these studies, the recent one showed a surpassing Kappa value of 0.72 with the U-Net model compared with the results given by the expert pathologists (Kappa: 0.640) [47].

#### 2.2.4. Prognosis Prediction

Only three models have been developed for prognosis prediction [20,45,49]. Bychkov et al. employed 420 tissue microarray (TMA) WSIs to predict five-year disease-specific survival of patients and achieved an AUC of 0.69 [49]. Kather et al. used more than 1000 WSIs from three institutions to predict the prognosis using histological slides and achieved up to 99% accuracy [20], and another model by the same author trained the ResNet model to detect microsatellite instability (MSI) directly from histological images and achieved AUC of 0.77 for The Cancer Genome Atlas (TCGA)–formalin-fixed paraffin-embedded samples (DX), and 0.84 for TCGA-snap-frozen samples dataset (KR) [45].

## 3. Discussion

In this study, we found that most studies thus far are in the elementary stage with datasets of a small scale with low information, although the results are quite promising with acceptable performance. In our review, we identified several challenges in the appropriate adoption of AI for the pathological diagnosis of CRC.

### 3.1. Challenges in Pathological Diagnosis of CRC

#### 3.1.1. Small Tissue Artifacts

Pathological diagnosis of the colonoscopy-biopsied CRC tissue is a challenging task because of the small size of the tissue sample and the squeezing and cauterization artifacts that occur during sampling [51]. These artifacts within a small tissue sample can lead to the interpretational error in benign versus malignant.

#### 3.1.2. Regenerative Atypia

It is challenging to differentiate the premalignant process such as adenomatous polyps from the changes within the reparative process. Colonic mucosa is one of the most highly regenerative tissues in the human body owing to its frequent exposure to temporary infection or inflammation. There are also several chronic inflammatory conditions such as inflammatory bowel disease including Crohn’s disease and ulcerative colitis. In these clinical situations, regenerative activity is increased for a long time, and nuclear and structural atypia is often found, which leads to misdiagnosis of adenoma [51]. Making an accurate diagnosis can be very challenging even for expert pathologists in small biopsy samples.

#### 3.1.3. Inter-Observer Variation in Adenoma Grading and Subtype Classification

The classification system of premalignant neoplasms of CRC is still under debate among all pathologists worldwide. Generally, the Vienna and Japanese classification systems are being widely used by Western and Eastern pathologists, respectively [6,7]. However, there is a diagnostic difference between the two systems. Most Western pathologists use tumor invasion as a diagnostic feature for carcinoma, while Eastern pathologists rely on glandular and nuclear features of the tumor cells [6,7]. A previous study showed that the histological concordance between both classifications was 15% (three out of 20 cases) [8]. In 2000, an international panel of expert pathologists proposed new classification criteria to resolve these discrepancies and classify colorectal neoplasia into five types. Category 1 and 2 tumors are negative for neoplasia/dysplasia. Category 3 includes low-grade adenoma/dysplasia, Category 4 includes high-grade adenoma/dysplasia, and Category 5 involves invasive lesions carrying a higher risk of metastases [52]. However, as the invasion beyond the basement membrane is difficult to define, neither Western nor Japanese pathologists were able to differentiate high-grade adenoma and intramucosal carcinoma in a reliable manner in many cases [8,52]. Hence, there is a need for robust software that can increase the reliability of diagnosis.

#### 3.1.4. Importance of Tumor Microenvironment in CRCs

The tumor microenvironment (TME) has become the key to understanding the tumorigenesis and tumor progression of CRC. TME refers to the mutual interaction between tumor cells, immune cells, blood vessels, extracellular matrix proteins, etc. [53]. It is well known that CRCs with tumor-infiltrating lymphocytes (TILs) have better overall and disease-free survival and are closely related to MSI and CpG island methylator phenotype (CIMP) [54,55,56]. Recently, these facts have been highlighted by the emergence of immune checkpoint inhibitors such as PD-L1 (programed death-ligand 1) inhibitors. Various ongoing trials found improved progression-free survival in patients with MSI-high CRC [57].

However, the quantitative characterization of TME is a very challenging task for the pathologist to differentiate precisely due to heterogeneity of cells. Conventionally, the expert pathologist uses a three-tier system, namely, mild, moderate, and severe, to measure the TME; however, there are still inter-observer and intra-observer variations and reproducibility issues between pathologists [58,59]. Hence, there is an urgent need for automatic recognition software that can precisely quantify the TME.

#### 3.1.5. Prognostic Prediction in CRCs

Currently, evaluation of the patient’s prognosis largely depends on the tumor-node-metastasis (TNM) system of the American Joint Committee on Cancer (AJCC) and is the best predictor used to guide therapy [60]. However, in stage II patients, for example, the sub-stage (IIA, IIB, and IIC) depends on only the depth of tumor invasion (T stage, T3, T4a, T4b, respectively) but not on lymph node metastasis (N stage, N0). Although the current standard treatment for stage II patients is surgery without neoadjuvant or adjuvant therapies, some patients experience tumor recurrence probably due to potential lymph node metastasis, and the current TNM staging system cannot identify these patients [61,62]. This represents the need for innovative staging system such as computerized techniques using AI.

Recently, one of the most popular fields in pathological diagnosis is AI that directly predicts patient prognosis and response to treatment from histopathological slides to facilitate precision medicine [63]. Up until now, although certain histologic features such as tumor grade and subtype have been used significant prognostic factors so far, direct linking of CRC images with survival outcomes remains largely unexplored. [63].

### 3.2. Application of Deep Learning Models in CRC Pathological Diagnosis

#### 3.2.1. Gland Segmentation

Most cancers in the human body arise from two kinds of cells: Epithelial cells, leading to carcinomas; and stromal cells, leading to sarcomas. For this reason, differentiating epithelial tissue (mostly glands) from stroma is a fundamental step in the process of the pathological diagnosis [64]. Approximately 90–95% of carcinomas may arise from glands such as salivary glands, breast, prostate, upper and lower GI tracts, sweat glands, exocrine pancreas, and the biliary tract [64]. In these gland-derived carcinomas, the so-called adenocarcinomas, the structure, shape, and size of the glands are very important features for grading [64]. The GlaS challenge in 2015 was held to solve this problem. In total, 110 teams participated in the challenge and developed an optimal segmentation model using a dataset of 165 patch images with annotation from 16 CRC WSIs (Warwick QU dataset, Munich, Germany, [22]. The dataset consists of a training part (37 benign and 48 malignant images), a test part A (33 benign and 27 malignant images), and a test part B (four benign and 16 malignant images) [22]. As a result, the CUMedVision model developed by Chen et al. ranked first in the challenge [21,22]. In this study, a fully trained convolutional network-based deep contour-aware network (FCN-DCAN) model was proposed that produces the probability map directly with a single forward propagation. One of the main advantages of their model is that it can be easily generalized to biopsy samples with different types of histopathological grades, including malignant and nonmalignant cases. In addition, their model can segment the gland objects and contours simultaneously instead of an independent task [21].

**Figure 4:**
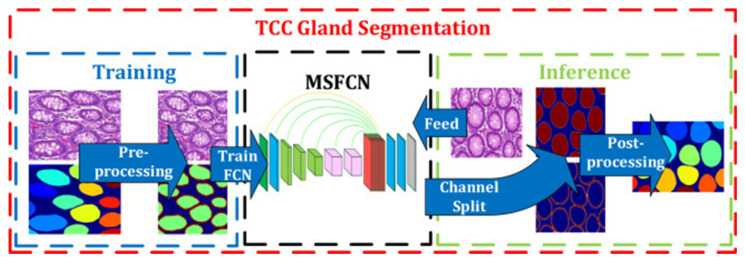
Example of colon cancer segmentation. The flow chart of the three-class classification multi-scale fully convolutional network model. The left side represents the training process and the right side represents the inference. The glands are labeled by different colors at last. Figure 2 in Ding et al. [33]. Reprinted with permission from the authors [33]. Copyright (2019), Neurocomputing published by ELSEVIER.

After the GlaS challenge, most studies tried to enhance the design of the model, and some of the studies used additional datasets for better performance. In our comparison, the model proposed by Ding et al. in 2019 showed the best performance of all, thus far, according to the rank-sum analysis of three parameters (Table 3). They added a colorectal adenocarcinoma gland (CRAG) dataset for training and developed a three-class classification multi-scale fully convolutional network model (TCC-MSFCN) (Figure 4) [33]. The model showed better performance owing to its design by combining features such as dilated convolution, high-resolution branch, and residual structure. The foremost advantage of this model is that the MSFCN model can also segment the glands with a variable dataset, and TCC can precisely differentiate very closely spaced glands. In addition, they performed a series of experiments with a variable dataset to check the robustness of their combined features [33].

#### 3.2.2. Tumor Classification

Several deep learning models have also been developed to reduce the intra- or inter-observer variation when classifying tumors into various subtypes. In a recent study conducted by a Dutch group about intra- and inter-observer variations on the diagnosis of 66 slides of stage IV CRC, there was moderate agreement among 12 pathologists (kappa = 0.50) and a substantial intra-observer agreement among eight pathologists (kappa = 0.71), which represent the current subjectivity of the pathological diagnosis process and the need for more reproducible interpretations [65].

**Figure 5:**
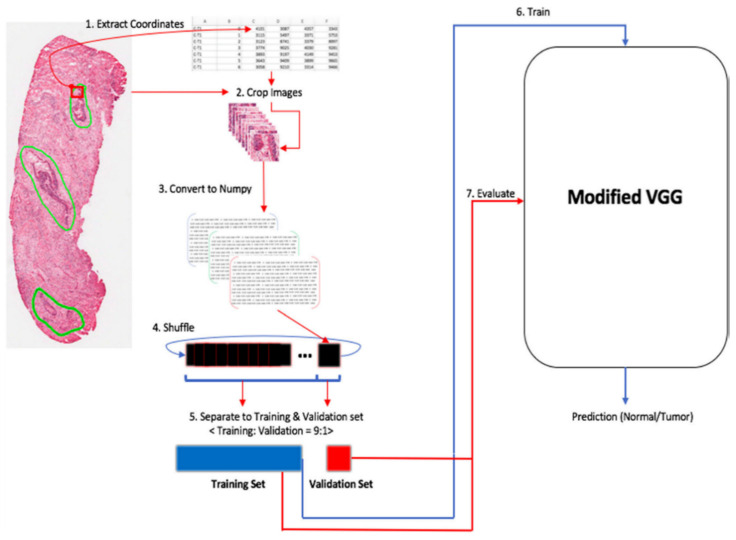
Example of histological classification of CRC. The classification workflow. First, the coordinates of the region of interest are extracted from the original images. Using those images and the extracted coordinates, images are cropped to 256 × 256 pixels. Images are the converted to numpy format for efficiency, shuffled, and separated to training and validation sets at a ratio of 9:1. Finally, the training set is fed to the modified VGG model for training; after each epoch, the training and validation sets’ prediction evaluated. Figure 2 in Yoon et al. [41]. Reprinted with permission from the authors [41]. Copyright (2018), Journal of Digital Imaging published by Springer Link.

Although all eight studies in this category showed very high-performance results, they should be interpreted with caution, considering some limitations of these studies. In most studies, cropped or patch images rather than WSIs were used for training (Figure 5). The cropped images from one WSI include similar histological features because they are all from one CRC patient, even though thousands of cropped images were used. In terms of increasing the generalization of the algorithm, millions of patches from fewer than dozen WSIs cannot be compared with thousands of WSIs. In this sense, only one study conducted by Lizuka et al. that used 4036 CRC WSIs, seems to be noteworthy [44].

In terms of the quality of annotation as well as the scale of training data, most studies did not report enough information on the annotation process, such as how many pathologists were involved, how the ground truth data for validation were prepared, and how the disagreement between pathologists were resolved.

Regarding the validity, it is generally recommended to prepare the training data annotation with many pathologists as possible to minimize inter-observer variation. In addition, it is also recommended to perform external cross-validation using datasets from external institutions or publicly available data such as The Cancer Genome Atlas (TCGA) or GTEx Histological Images [66]. In this context, only Kather et al. study used external validation dataset from the Darmkrebs Chancen der Verhütung durch Screening (DACHS) study to validate the performance of their model [45].

Another factor that precludes direct comparison of the model is the existence of a different classification system for precancerous lesions. The classification system in each study was made arbitrarily from two to six classes, which is also due to the difference in interpretational opinions between the Japanese and Vienna systems.

Thus, any conclusions based on these records involve a significant level of uncertainty. More studies with a higher number of datasets with more qualified annotations with appropriate validation are required for future studies.

#### 3.2.3. Tumor Microenvironment Analysis

**Figure 6:**
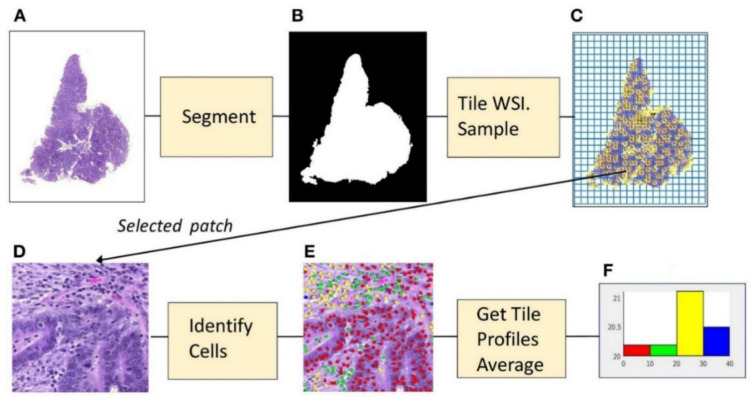
Example of microenvironment analysis from CRC slides. (**A**) Histopathology image. (**B**) Foreground mask. (**C**) Tiling, with sampled patches. (**D**) Sampled patch. (**E**) Cells identified by the algorithm. (**F**) Histogram of cell frequencies. Figure 1 in Shapcott et al. [48], Reprinted with permission from the authors [48]. Copyright (2019), the Frontiers in Bioengineering and Biotechnology published by Frontier.

In most TME studies, a combination of classification and segmentation was used, sometimes with the adoption of IHC slides. Shapott et al. designed a CNN model that classified epithelial, inflammatory, fibroblast, and other cells (Figure 6). The WSIs were segmented into the background (no tissue present) and foreground (different tissue present), and the images were cropped into 500 pixels in width for input, achieving an average of 65% detection accuracy and 76% classification accuracy [48]. Interestingly, they also found a significant association between increased fibroblast cells and the presence of venous invasion, metastasis, lymphatic invasion, and residual tumor [48]. In another study by Siderska-Chadaj et al. used 28 IHC WSIs to train the CNN-based models that can automatically detect and differentiate T lymphocytes using CD3 and CD8 IHC. It showed good accuracy with reasonable robustness [47].

#### 3.2.4. Prognosis Prediction 

**Figure 7:**
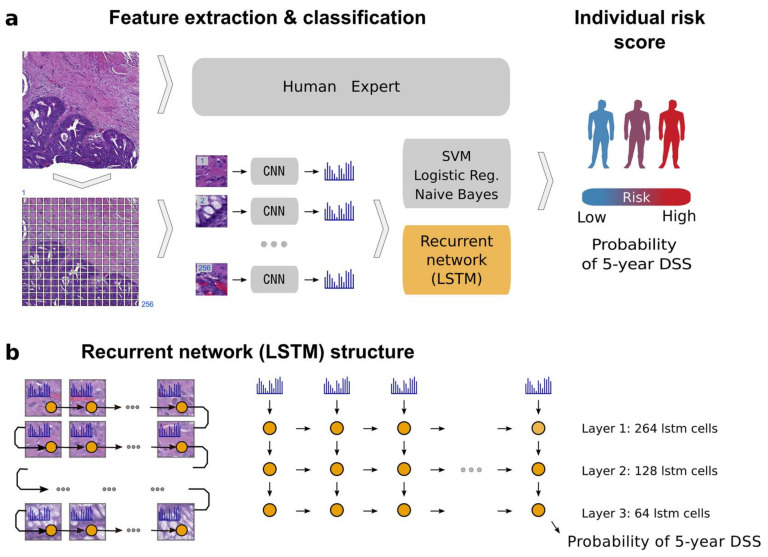
Example of patient prognosis of CRC patients. Overview of the image analysis pipeline and long short-term memory (LSTM) prognostic model. Images of tissue microarray (TMA) spots are characterized by a pre-trained convolutional neural network (VGG-16) in a tile-wise manner. The VGG-16 network produces a high-dimensional feature vector for each tile from an input image. These features then serve as inputs for classifiers trained to predict five-year disease-specific survival (DSS) (**a**). The long short-term memory (LSTM) network slides through the entire image of the tissue microarray spot to jointly summarize observed image tiles and predict the patient risk score (**b**). Figure 1 in Bychkov et al. [49], copyright 2018 the author(s), reprinted from the Scientific Reports published by Nature. This is an open access articles under the term of the Creative Commons attribution-Non Commercial-NoDerivs License.

Prognosis prediction based on H&E images is still in its elementary stage with many limitations. In both studies in this category, we found that the training data were relatively limited to obtain sufficient generalization. For example, Bychkov et al. used 420 TMA WSIs (Figure 7). Although it showed a better performance in the prediction of disease-specific survival with a higher AUC of 0.69 than human experts (0.57–0.58), the possibility of overfitting cannot be ruled out given the small sample size [49]. In another study conducted by Kather et al., the model classified nine types of tissue, namely, adipose, background, debris, lymphocytes, mucus, smooth muscle, normal mucosa, stroma, and cancer epithelium. Using the five tissue classes that were found to be associated with poor prognosis, and they developed a prognostic metric called “deep stroma score,” which is independently associated with recurrence-free survival (*p* = 0.0004) and overall survival (*p* = 0.008) [20]. Furthermore, in another study performed by the German group, 154,302 tiles were used that directly showed a direct correlation of MSIness (Fraction of MSI-predicted patches) with interferon-γ signature and PD-L1 expression. Moreover, using DACHS cohort dataset showed the worse overall survival (*p* = 0.0207) in high-MSIness group [45].

Although limited, there is a possibility that pathologic images using deep learning can predict the therapeutic effects of chemotherapy or molecule-targeted therapy. More studies on this subject need to be performed in the future.

### 3.3. Limitation of This Study

In this review, we compared GlaS and post-GlaS models using three parameters. However, it should be interpreted carefully because there are several issues according to the metrics that has been used in the comparison of model performance and the handling method of such international challenges or competitions. Maier-Hein et al. mentioned, in their review paper about AI competitions, that slight changes in these metrics can shift the rankings so drastically that the lowest-ranking entry became the winner [67]. Consequently, they claimed that when releasing the results of each competition entry, it is important to reveal the details of how the metrics were evaluated [67]. In addition, as the classical performance metrics are comparative parameters that reflect one-dimensional performance of the model, there has been a demand for development of novel parameters. The performance of the deep-learning algorithm should be evaluated more comprehensively using the variety of new parameters.

## 4. Materials and Methods

### 4.1. Literature Search

This study was approved by the Institutional Review Board of the Catholic University of Korea, College of Medicine (SC20ZISE0050). We searched major electronic databases for relevant articles published in medical journals between January 2000, and January 2020, with abstracts written in English. The databases included MEDLINE (PubMed), Cochrane Library, and EMBASE databases. The query terms used were “colorectal neoplasm,” “gland segmentation,” “colonic neoplasms,” “pathology,” “histology,” “artificial intelligence,” “deep learning,” “image processing,” “computer-assisted,” “machine learning,” “colorectal histological deep learning.” Among the searched articles, non-English-language articles and articles without available full texts were excluded. Further references were obtained by cross-referencing the key articles. The records were retrieved and managed with EndNote X9 (Bld 10136, Thomson Reuters, New York, NY, USA).

### 4.2. Study Selection, Reviewing, and Data Retrieval

The process of study selection and review is depicted in Figure 1. After the initial search, duplicate records were removed from the results. Then, the titles and abstracts of the studies were screened by two independent reviewers (N.T. and Y.C.). Case reports, reviews, letters, conference proceedings, and posters were excluded. Original studies about deep learning in pathological image analysis were included for full-text review. Additionally, the cited references in each study were manually searched and reviewed to identify any additional relevant studies.

## 5. Conclusions

Overall, our study concluded that artificial intelligence showed promising results in terms of accuracy for diagnosing CRC. However, the scale and quality of the training and validation datasets of most of the studies were relatively limited to apply this technique in clinical practice. In addition, external cross-validation, especially in tumor classification, is required. Therefore, future studies with a larger number of datasets with high-quality annotations and external cross-validation are required for routine practice-level validation. Now, we are one step behind the clinical application of AI in pathology to replace the conventional microscopic diagnosis.

## Figures and Tables

**Table 1:** The performances of the gland segmentation models using Gland Segmentation in Colon Histology Images (GlaS) challenge dataset (165 images Warwick-Qu dataset and CRAG data set). The models that participated GlaS challenge are designated with * and the models that were published using GlaS dataset only are designated with ǂ. The performance of each model is evaluated based on the F1-score (accuracy of the detection of individual glands), Object Dice (agreement or similarity between two sets of samples), and Object Hausdorff (boundary-based similarity between glands and their corresponding segmentation). Part A means the off-site dataset and Part B means the on-site dataset. The best performance in the respective part appears bold in the table.

Model No.	Author/Team	Dataset	Base Model	Performance Metrics						Year	Country	Ref.
				F1-Score		Object Dice		Object Hausdorff				
				Part A	Part B	Part A	Part B	Part A	Part B			
1 *	CUMed Vision2	WQD	DCAN	0.912	0.716	0.897	0.781	45.4	160.3	2015	Hong Kong	[21]
2 *	ExB1	WQD	Two path CNN	0.891	0.703	0.882	0.786	57.4	145.6	2015	Germany	[22]
3 *	ExB3	WQD	Two path CNN	0.896	0.719	0.886	0.765	57.4	159.9	2015	Germany	[22]
4 *	Freiberg2	WQD	U-Net	0.87	0.695	0.876	0.786	57.1	148.5	2015	Germany	[22]
5 *	CUMed Vision1	WQD	FCN	0.868	0.769	0.867	0.8	74.6	153.6	2015	Hong Kong	[21]
6 *	ExB2	WQD	Two path CNN	0.892	0.686	0.884	0.754	54.8	187.4	2015	Germany	[22]
7 *	Freiburg1	WQD	U-Net	0.834	0.605	0.875	0.783	57.2	146.6	2015	Germany	[22]
8 *	CVML	WQD	CNN	0.652	0.541	0.644	0.654	155.4	176.2	2015	UK	[22]
9 *	LIB	WQD	K-means/naïve Bayesian	0.777	0.306	0.781	0.617	112.7	190.4	2015	France	[22]
10 *	Vision4GlaS	WQD	Object-Net/Separator-Net	0.635	0.527	0.737	0.61	107.5	210.1	2015	Austria	[22]
**11 ǂ**	BenTaieb	WQD	FCN+Smoothness+Topology	NA	NA	0.80 ± 0.12		NA	NA	2016	Canada	[23]
**12 ǂ**	Li	85 WQD	CNN+HC-SVM	NA	NA	0.87 ± 0.08		NA	NA	2016	UK	[24]
**13 ǂ**	Yang	WQD	FCN	0.921	**0.855**	0.904	**0.858**	44.7	97.0	2017	USA	[25]
**14 ǂ**	Kainz	WQD	Object-Net	0.670	0.570	0.70	0.620	137.4	216.4	2017	Austria	[26]
			Separator-Net	0.680	0.610	0.750	0.650	103.5	187.8			
**15 ǂ**	Zhang	WQD	DAN	0.916	**0.855**	0.903	0.838	45.3	105.0	2017	USA	[27]
**16 ǂ**	Xu	WQD	FCN/DCAN/RPN/CNN	0.893	0.843	0.908	0.833	44.1	116.8	2017	China	[28]
**17 ǂ**	Graham	WQD /16 CRAG WSIs	MILD-Net	0.914	0.844	**0.913**	0.836	41.5	105.9	2018	UK	[29]
**18 ǂ**	Yan	WQD	Holistically-nested networks	**0.924**	0.844	0.902	0.840	49.9	106.1	2018	Hong Kong	[30]
**19 ǂ**	Manivannan	WQD	FCN	0.892	0.801	0.887	0.853	51.2	**87.0**	2018	UK	[31]
**20 ǂ**	Tang	WQD	Segnet	NA	NA	0.882	0.836	106.6	102.6	2018	China	[32]
**21 ǂ**	Ding	WQD/213 CRAG images	TCC-MSFCN	0.914	0.85	**0.913**	**0.858**	**39.8**	93.2	2019	China	[33]
**22 ǂ**	Raza	WQD	MIMO-Net	0.913	0.724	0.906	0.785	49.2	134.0	2019	UK	[34]
**23 ǂ**	Liu	WQD	Wavelet Scattering Network	0.874	0.71	0.875	0.791	56.6	146.6	2019	China	[35]
**24 ǂ**	Binder	WQD	U-Net	NA	NA	0.920		11.0		2019	France	[36]
**25 ǂ**	Khvostikov	WQD/20 PATH- DT-MSU images	U-Net	NA	NA	0:880		NA	NA	2019	Russia	[37]

WQD, 165 images Warwick-Qu dataset; DCAN, deep contour-aware network; CNN, convolution neural network; FCN, fully convolutional networks; CRF-RNN, conditional random fields- recurrent neural networks; HC-SVM, handcrafted features-support vector machine; DAN, deep adversarial network; RPN, region proposal network; MILD, minimal information loss dilated network; CRAG, Colorectal Adenocarcinoma Gland dataset; TC-MSFCN, three-class classification multi-scale fully convolutional network.

**Table 2:** Characteristics of the deep-learning models according to tumor classification, tumor microenvironment analysis, and prognosis prediction of colorectal cancers using pathologic image analysis.

Model No.	Feature	Task	Dataset	External Cross-Validation	Base Model	Performance	Author/Team	Year	Country	Ref.
**1**	**Tumor Classification**	6 classes (cancer subtypes): NL/ADC/MC/SC/PC/CCTA	717 patches	Not done	AlexNet	Accuracy—97.5%	Xu	2017	China	[38]
**2**		5 classes (polyp subtypes):	2074 patches 936 WSI	Not done	ResNet	Accuracy—93.0%	Korbar	2017	USA	[39]
		NL/HP/SSP/TSA/TA/TVA-VA,								
**3**		3 classes: NL/AD/ADC	30 multispectral image patches	Not done	CNN	Accuracy—99.2%	Haj-Hassan	2017	France	[40]
**4**		2 classes: NL/Tumor	57 WSI (10,280 patches)	Not done	VGG	Accuracy—93.5%,	Yoon	2018	South Korea	[41]
						Sensitivity—95.1%				
						Specificity—92.8%				
**5**		3 classes: NL/AD/ADC	27 WSI (13,500 patches)	Not done	VGG16	Accuracy—96 %	Ponzio	2018	Italy	[42]
**6**		4 classes: NL/HP/AD/ADC	393 WSI	Not done	CNN	Accuracy—80%	Sena	2019	Italy	[43]
			(12,565 patches)							
**7**		3 classes: NL/AD/ADC	4036 WSI	Not done	CNN/RNN	AUCs—0.96 (ADC)	Iizuka	2020	Japan	[44]
						0.99 (AD)				
**8.**		2 classes: NL/Tumor	94 WSI,	Done using 378 DACHS data	ResNet18	AUC > 0.99	Kather	2019	Germany	[45]
			370 TCGA-KR,							
			(60,894 patches)							
			378 TCGA-DX,							
			(93,408 patches)							
**9**	**Tumor Microenvironment Analysis**	Classification, Segmentation and Detection: EC/IC/FC/MC	21,135 patches	Not done	DCRN/R2U-Net	**Classification**	Alom	2018	USA	[46]
						F1-score—0.81				
						AUC—0.96				
						Accuracy—91.1%				
						**Segmentation**				
						Accuracy—92.1%				
						**Detection**				
						F1 score—0.831				
**10**		Detection of immune cell CD3^+^, CD8^+^	28 WSI IHC	Not done	FCN/LSM/U-Net	FI score—0.80 Sensitivity—74.0% Precision—86	Swiderska-Chadaj	2019	Netherland	[47]
**11**		Detection and classification EC/IC/FC/MC	853 patches & 142 TCGA images	Not done	CNN	**Detection** Accuracy—65% **Classification** Accuracy—76 %	Shapcott	2019	UK	[48]
**12**		Classification of 9 cell types ADI, BAC, DEB, LYM, MUC, SM, NL, SC and EC	86 WSI (100,000) NCT&UMM	Not done	VGG19	Accuracy—94–99%	Kather	2019	Germany	[20]
**13**	**Prognosis Prediction**	5-year disease-specific survival	420 TMA	Not done	LSTM	AUC—0.69	Bychkov	2018	Finland	[49]
**14**		Survival predictions	25 DACHS WSI	Not done	VGG19	Accuracy—94–99%	Kather	2019	Germany	[20]
			862 TCGA WSI							
			409 DACHS WSI							
**15**		MSI predictions	360 TCGA- DX (93,408 patches) 378 TCGA- KR (60,894 patches)	Done using 378 DACHS data	ResNet18	AUC TCGA-DX—0.77 TCGA-KR—0.84	Kather	2019	Germany	[45]

NL, normal mucosa; ADC, adenocarcinoma; MC, mucinous carcinoma; SC, serrated carcinoma; PC, papillary carcinoma; CCTA, cribriform comedo-type adenocarcinoma; HP, hyperplastic polyp; SSP, sessile serrated polyp; TSA, traditional serrated adenoma; TA, tubular adenoma; TVA, tubulovillous adenoma; VA, villous adenoma; WSI, whole slide images; TCGA, The Cancer Genome Atlas; DACHs, Darmkrebs Chancen der Verhütung durch Screening; ResNet, residual network architecture; VGG, visual geometry group; AD, adenoma; CNN, convolution neural network; RNN, recurrent neural network; AUC, area under the curve; IHC, immunohistochemistry; TMA, tissue microarray; EC, epithelial cell; SC, stromal cells; DCNN, deep convolution neural network; MCC, Matthew correlation coefficient; MC, miscellaneous; DCRN, densely connected recurrent convolutional network; R2U-Net, recurrent residual U-Net; FCN, fully convolutional networks; IC, inflammatory cell; FC, fibroblast cell; ADI, adipose; BAC, background; DEB, debris; LYM, lymphocytes; MUC, mucus; SM, smooth muscle; LSM, locality-sensitive method; LSTM, long short-term memory; MSI, microsatellite instability.

**Table 3:** The rank-sum analysis of the performance metrics of the gland segmentation studies using GlaS challenge dataset. The best performance in the respective part appears bold in the table.

Model No.	Author/Team	Performance Metrics												Rank-Sum	Rank-Sum Rank	Year	Ref.
		F1-Score				Object Dice				Object Hausdorff							
		Part A	Rank	Part B	Rank	Part A	Rank	Part B	Rank	Part B	Rank	Part B	Rank				
**19 ǂ**	Ding	0.914	4	0.850	3	**0.913**	1	**0.858**	1	**39.8**	1	93.2	2	12	**1**	2019	[33]
**11 ǂ**	Yang	0.921	2	**0.855**	1	0.904	5	**0.858**	1	44.7	4	97.0	3	16	**2**	2017	[25]
**15 ǂ**	Graham	0.914	4	0.844	4	**0.913**	1	0.836	6	41.5	2	105.9	5	22	**3**	2018	[29]
**13 ǂ**	Zhang	0.916	3	**0.855**	1	0.903	6	0.838	5	45.3	5	105.0	4	24	**4**	2017	[27]
**16 ǂ**	Yan	**0.924**	1	0.844	4	0.902	7	0.840	4	49.9	8	106.1	6	30	**5**	2018	[30]
**14 ǂ**	Xu	0.893	9	0.843	6	0.908	3	0.833	7	44.1	3	116.8	7	35	**6**	2017	[28]
**17 ǂ**	Manivannan	0.892	10	0.801	7	0.887	9	0.853	3	51.2	9	**87.0**	1	39	**7**	2018	[31]
**20 ǂ**	Raza	0.913	6	0.724	9	0.906	4	0.785	12	49.2	7	134.0	8	46	**8**	2019	[34]
1 *	CUMedVision2	0.912	7	0.716	11	0.897	8	0.781	14	45.4	6	160.3	15	61	**9**	2015	[21]
**21 ǂ**	Liu	0.874	13	0.710	12	0.875	14	0.791	9	56.6	11	146.6	10	69	**10**	2019	[35]
2 *	ExB1	0.891	12	0.703	13	0.882	12	0.786	10	57.4	15	145.6	9	71	**11**	2015	[22]
3 *	ExB3	0.896	8	0.719	10	0.886	10	0.765	15	57.4	14	159.9	14	71	**11**	2015	[22]
4 *	Freiberg2	0.870	14	0.695	14	0.876	13	0.786	10	57.1	12	148.5	12	75	**13**	2015	[22]
5 *	CUMedVision1	0.868	15	0.769	8	0.867	16	0.800	8	74.6	16	153.6	13	76	**14**	2015	[21]
6 *	ExB2	0.892	10	0.686	15	0.884	11	0.754	16	54.8	10	187.4	17	79	**15**	2015	[22]
7 *	Freiburg1	0.834	16	0.605	17	0.875	14	0.783	13	57.2	13	146.6	10	83	**16**	2015	[22]
**12 ǂ**	Kainz	0.680	18	0.610	16	0.750	18	0.650	18	103.5	17	187.8	18	105	**17**	2017	[26]
8 *	CVML	0.652	19	0.541	18	0.644	20	0.654	17	155.4	20	176.2	16	110	**18**	2015	[22]
9 *	LIB	0.777	17	0.306	20	0.781	17	0.617	19	112.7	19	190.4	19	111	**19**	2015	[22]
10 *	Vision4GlaS	0.635	20	0.527	19	0.737	19	0.610	20	107.5	18	210.1	20	116	**20**	2015	[22]
